# An Examination of the Meikirch Model’s Multidimensional Definition of Health and Aging

**DOI:** 10.1093/geroni/igaa057.1200

**Published:** 2020-12-16

**Authors:** Hillary Dorman, Rebecca Allen, Patricia Parmelee, Lisa McGuire

**Affiliations:** 1 University of Alabama, Tuscaloosa, Alabama, United States; 2 Alabama Research Institute on Aging, Tuscaloosa, Alabama, United States; 3 Centers for Disease Control and Prevention, Atlanta, Georgia, United States

## Abstract

The present study used the CDC’s 2017 Behavioral Risk Factor Surveillance System and Structural Equation Modeling (SEM) to test the Meikirch model’s theoretical definition of health and aging. This biopsychosocial and ecological framework considers the dynamic interplay between an individual’s biology, health behavior and health potential, social surroundings, physical environment, and demands of living on both mental and physical health outcomes. A total sample of 96,568 adults were included with a mean age of 66.05 years (SD = 9.91). Individuals were classified in the following age groups: 43% middle-aged (45-64 years), 33% young-old (65-74 years), and 25% old-old adults (75+ years). The sample was largely female (61%), Non-Hispanic White (86%), and urban (67%). A CFA and WLSMV estimation was used to assess each latent construct. The overall SEM model was found to be a good fit (RMSEA=0.05, CFI=0.94, TLI=0.90), explained a significant portion of the variance in the health outcome (65%), and appeared to mimic prior literature on health variable relationships. An important finding within this study suggests the significant relationship between education, personally acquired potential, and health outcome variables. Underscoring health education and health literacy interventions may positively promote a person’s health behavior, resource access, and health outcomes across the lifespan. The Meikirch model can be used as a framework in public health interventions to better understand health adaptation, as well as behavioral risks and systematic hurdles. Overall, the study emphasizes how understanding health is not exclusively an individual hurdle to tackle, but a communal goal.

